# Scaling Tableting Processes from Compaction Simulator to Rotary Presses—Mind the Sub-Processes

**DOI:** 10.3390/pharmaceutics12040310

**Published:** 2020-03-31

**Authors:** Isabell Wünsch, Irene Friesen, Daniel Puckhaber, Thomas Schlegel, Jan Henrik Finke

**Affiliations:** 1 Institute for Particle Technology, TU Braunschweig, Volkmaroder Str. 5, 38104 Braunschweig, Germany; i.wuensch@tu-braunschweig.de (I.W.); irene.friesen@novartis.com (I.F.); d.puckhaber@tu-braunschweig.de (D.P.); 2 Center of Pharmaceutical Engineering—PVZ, TU Braunschweig, Franz-Liszt-Str. 35A, 38106 Braunschweig, Germany; 3 KORSCH AG, Breitenbachstr. 1, 13509 Berlin, Germany; Thomas.Schlegel@korsch.de

**Keywords:** compaction simulation, scaling of tableting processes, lubrication, shear number, powder blends

## Abstract

Compaction simulators are frequently used in the formulation and process development of tablets, bringing about the advantages of flexibility, low material consumption, and high instrumentation to generate the most possible process understanding. However, their capability of resembling general aspects of rotary press compaction and their precision in simulating or mimicking sub-processes such as feeding and filling need to be systematically studied. The effect of material deformation behavior, blend composition, and feeding on tensile strength and simulation precision as compared with rotary presses of different scales is evaluated in this study. Generally, good simulation performance was found for the studied compaction simulator. Compaction profile-sensitivity was demonstrated for highly visco-plastic materials while shear-sensitivity in feeding was demonstrated for lubricated blends of ductile particles. Strategies for the compensation of both in compaction simulator experiments are presented by careful investigation of the compaction stress over time profiles and introduction of a compaction simulator-adapted shear number approach to account for differences in layout and operation mode between compaction simulator and rotary press, respectively. These approaches support the general aim of this study to provide a more straightforward determination of scaling process parameters between rotary press and compaction simulator and facilitate a quicker and more reliable process transfer.

## 1. Introduction

Tablets are the most frequently produced and applied dosage form. Nonetheless, their production process is not fully mechanistically understood, yet. Accordingly, formulation and process development of tablet production mostly rely on empirical trials and the practical knowledge of individuals. Additionally, results at lab scale are often not directly transferrable to production scale due to different filling regimes, compaction profiles, and specific velocities within the machine setups [1,2,3]. To overcome these hurdles and to enable formulation and process development at small scale with a low consumption of material, compaction simulators were engineered [4]. They try to circumvent challenges of different construction approaches and they are feasible to address formulation and process issues more flexibly than common tablet presses, i.e., rotary presses and eccentric presses. In general, compaction simulators compress bulk materials in a way that is comparable to industrially applied tableting and compacting processes such as roller compaction. To this end, a single compression station commonly equipped with a pair of punches and a die in a format, that is also used in industrial tableting processes, is provided. The punches are driven pneumatically, hydraulically, or by electric motors and in most cases controlled by a computer program. Compared to rotary presses, the die remains stationary and the feed frame moves over it in order to fill it gravitationally driven or by means of paddle wheels.

The question remains, with which precision compaction simulation can capture and deal with the effects of the diversity of process parameters and material properties in tableting. Primary challenges of compaction simulators are to timely correctly resemble the punch movements and by that the compaction profiles of original rotary presses. Furthermore, it must be clarified to which extent and by means of which scaling methods, specific challenges that arise in different sub-processes additional to the compression phase such as feeding, filling, and ejection can generally be studied on a compaction simulator and be transferred back to rotary tablet presses.

Literature provides a number of studies applying compaction simulators as flexible and material-sparing devices for scientific studies and formulation development [5,6,7,8,9], such as studying the effect of process parameters on forces in the sub-process of tablet ejection [10,11]. Also, the applicability of compaction simulators to simulate and predict results of roller compaction trials are highlighted [12,13,14]. However, direct comparisons of tablet properties of compaction simulation experiments with original results of the respectively simulated rotary presses are very limited in the literature. Only Bourland and Mullarny provide a direct and comprehensive comparison of the tablet strength of tablets of a marketed product produced on two rotary presses and their dwell time equivalent simulation on the Presster mechanical compaction simulator [15], interpreting data from the work of Guntermann [16]. Tablet breaking force over main compression force (at the upper punch) is presented, displaying up to 20% lower breaking forces for the original rotary presses as compared with their simulations on the Presster. However, the compression data of the Presster, simulating either of both rotary presses, were in very good agreement to each other, hinting at the fact that sub-processes other than the compaction, defined by the compaction stress and the dwell time, influence the result of tableting simulation studies. Nofrerias et al. present work on the application of the compaction simulator Styl’One in formulation and process development of a Zidovudine formulation [17]. They display results of compaction simulator experiments, elucidating the influence of process parameters turret speed and pre-compression and claim that the found results are comparable to those of the simulated rotary presses. However, data for the rotary presses and, by that, a direct comparison of results is not provided.

Commonly, blends of multiple bulk solids are compressed to tablets in industrial applications. This makes the compression process more complex for homogeneous blends [7], but also brings along the risk that segregation [18], attrition [19,20], dispersion [21,22,23], and (over)mixing play a vital role for the quality of the final product. These mechanisms typically take place before the compression process in the sub-processes of blend preparation [22], feeding [24], and filling of the dies. These sub-processes are in most cases not focus of studies on compaction simulators. Accordingly, knowledge and models are needed to better capture the effects and scaling them down from rotary presses to compaction simulators to represent them in more detail on such scales. Lubricated powders are a special case as they are highly susceptible to shear forces exerted to them during the passage through sub-processes prior to compaction [25]. Especially the tensile strength of internally lubricated, ductile materials is strongly influenced by the shear history and models were deduced to describe the effects of blending [22] and shear in rotary press feed frames [26]. However, these models are not directly applicable to compaction simulators and their adaption will be discussed.

In this study, compaction simulation is challenged, systematically evaluating the transferability of product properties (i.e., tensile strength, porosity) from a compaction simulator to different scales of rotary presses for common pharmaceutical ingredients. Crucial process and machine parameters are identified and elucidated and correction strategies for such current challenges are described.

## 2. Materials and Methods

### 2.1. Materials

Microcrystalline cellulose (MCC; Vivapur^®^ 102, JRS Pharma, Rosenberg, Germany), anhydrous dicalcium phosphate (DCP; Emcompress^®^ anhydrous, JRS Pharma, Rosenberg, Germany), lactose (Tablettose^®^ 70, Meggle, Wasserburg am Inn, Germany), partially pregelatinised starch (starch; Superstarch^®^ 200, DFE Pharma, Goch, Germany), and magnesium stearate (MgSt; Magnesia, Lüneburg, Germany) were used as tableting excipients. Based on the interpretation of compression data, e.g., applying the model of Heckel [27], literature describes the deformation behavior of MCC, DCP, lactose, and starch as plastic/ductile, brittle/fractioning, intermediate (partially ductile and brittle), and visco-plastic (e.g., [28]), respectively.

### 2.2. Powder Blending

Excipients for compaction simulator and pilot scale rotary press studies were blended 15 min at 60 rpm in a lab scale cube blender (filling ratio 40%, capacity 3.5 L, AR403, Erweka, Langen, Germany). For production scale studies, powders were blended for 15 min at 21 rpm in a drum blender (filling ratio 40%, capacity 100 L, J. Engelsmann AG, Ludwigshafen am Rhein, Germany). To avoid high ejection forces and wear [29,30], pure DCP and lactose were mandatorily and MCC was optionally lubricated by admixing 1 wt.-% of MgSt.

### 2.3. Compaction Simulation

Lab scale compaction studies were performed with a Styl’One evolution compaction simulator (CS; Medel’Pharm, Beynost, France). It was equipped with 9.00 mm punches producing tablets of approx. 300 mg, 11.28 mm punches producing tablets of approx. 450 mg, or 14 mm punches producing tablets of approx. 725 mg. In either case, punches were round, flat-faced, and of euro D format. In-die data were evaluated applying the software ANALIS (Medel’Pharm, Beynost, France). Simulation profiles (punch displacement as a function of time) of the KORSCH XL 100 and KORSCH XL 400 were programmed based on geometric machine data (provided by KORSCH AG) using the software Profile’One (Medel’Pharm, Beynost, France). Compaction stresses of approx. 50, 150, and 300 MPa were applied and turret speeds in the range of 20 to 70 rpm were simulated. Ideal filling time *t*_f_ was calculated using Equation (1) as an estimation for the magnitude of the mean residence time in the feed frame of the CS. *t*_f_ assumes no dead volumes in the volume of the feed system *V*_R_ and a constant bulk density $\rho_{b}$ and it is calculated with the measured mass flow $\dot{m}.$

(1) $$t_{f}=\frac{V_{R}\cdot \rho_{b}}{\dot{m}}$$

### 2.4. Rotary Press Studies

Pilot and production scale investigations were performed on rotary presses KORSCH XL 100 and KORSCH XL 400 (XL100, XL400; both KORSCH AG, Berlin, Germany), equipped with 9 mm, 11.28 mm, or 14 mm flat-faced, round tooling (euro D format) and producing approx. 300, 450, or 725 mg tablets, respectively. The XL100 and XL400 were equipped with 4 and 29 dies and pairs of punches, respectively. Process parameters were in accordance with simulated parameter values (compaction stresses 50, 150, and 300 MPa, turret speed between 20 and 70 rpm). In-die data were evaluated with the software PharmaResearch^®^ (KORSCH AG, Berlin, Germany).

### 2.5. Tablet Characterisation

Geometric tablet parameters and breaking force were analyzed with tablet testing machines, applying either a MT50 (Dr. Schleuniger, Thun, Switzerland) or a UTS 4.1 (Kraemer Elektronik, Darmstadt, Germany). Tensile strength was calculated according to Fell and Newton [31] by applying Equation (2). Pore size distribution of selected samples was determined by mercury intrusion measurements (PoreMaster^®^ 60, Quantachrome, Odelzhausen, Germany). Ten tablets were tested and mean and standard deviation were calculated.

(2) $$\sigma_{t}=\frac{2\cdot F}{\pi \cdot h_{t}\cdot d_{t}}$$

where $\sigma_{t}$ is the tensile strength, F is the breaking force, $h_{t}$ is the height and $d_{t}$ the diameter of the tablet, respectively.

## 3. Results and Discussion

The use of compaction simulation in the development of dosage forms and production processes, especially the scale transfer to pilot and production scale rotary presses should be investigated as closely as possible to practice. Accordingly, the simulated rotary presses were implemented in the CS by straight forwardly using the supplied software that transfers geometrical machine information into punch movement profiles. This approach was chosen as this would be the way most probably applied in practice. Additionally, the parameters that are not readily scalable yet —i.e., the feed frame settings—were chosen to achieve convenient filling results in the first trials.

To elucidate the general feasibility of the transfer of compaction results from the CS to corresponding rotary presses, pure filler/binder materials and blends of the most differently deforming ductile MCC and brittle DCP are studied in Section 3.1. These are expected to be easily transferred as they do not display a highly time-dependent deformation behavior and are not prone to deagglomeration or overmixing as pure materials. Results for lactose and DCP, that need the addition of MgSt to avoid high ejection forces and wear, are accordingly excluded from this section.

Further investigations need to take the different sub-processes of the tableting process into account. Compaction simulation was laid-out to primarily mimic the compression phase. Accordingly, the effect of compaction simulation on a visco-plastic material will be evaluated in Section 3.2. However, also the filling of the die and in this context especially the agitation of the powder are of high interest and commonly less addressed in compaction simulation studies. The effect of the agitation in the feed frame and its scalability between CS and rotary press are studied regarding the functional excipient MgSt which tends to overlubrication, in Section 3.3.

### 3.1. General Simulation Capability and Filler Blends

**Figure 1:**
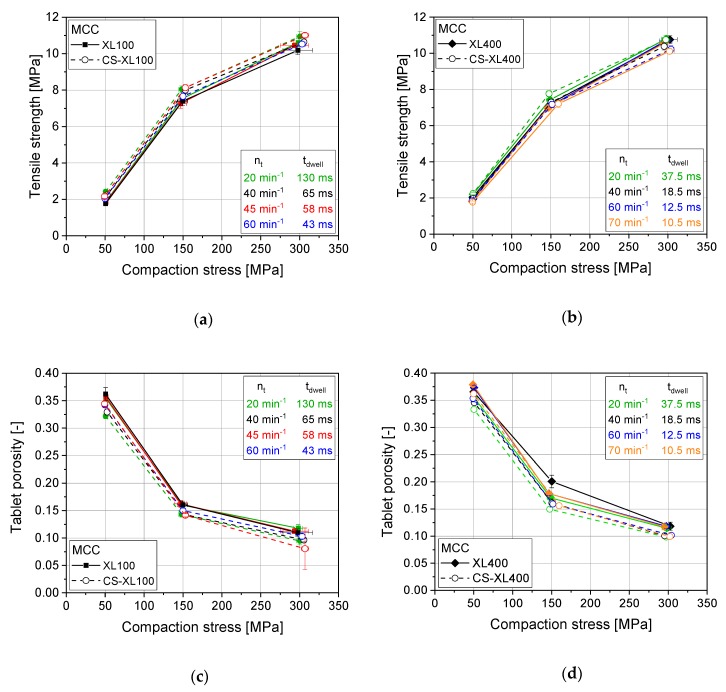
Tabletability profile for Microcrystalline cellulose on XL100 (**a**) and XL400 (**b**) and porosity as a function of compaction stress on XL100 (**c**) and XL400 (**d**) as well as for their simulated profiles (CS) applying 14 mm punches, respectively. *n* = 10.

For the pure excipients studied, a generally very good accordance between rotary press results and results of the CS mimicking the compaction profile of the respective machine was found regarding resulting tablet porosities and tensile strengths. On either scale of rotary press, pilot (XL100) or full production scale (XL400), results match for pure MCC as a very prominent example of excipients (Figure 1). However, only the compaction stresses and not the compaction speed are strength-determining for the final tablet made of MCC, as dwell times between 10 and 130 ms yield the same tensile strengths. Only slight trends towards lower porosities and higher tensile strengths for CS results for pure MCC are found as compared with rotary press results (Figure 1).

**Figure 2:**
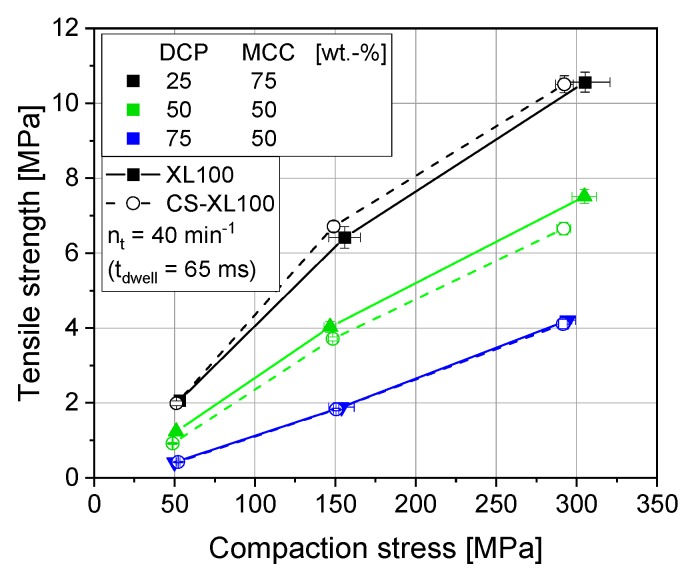
Influence of blend composition (anhydrous dicalcium phosphate/microcrystalline cellulose) on tabletability for XL100 and its simulated profile (CS) applying 11.28 mm punches, respectively. *n* = 10.

Studies of excipient mixtures of MCC and DCP display the drastic loss of tensile strength with rising DCP contents (Figure 2) due to its brittle deformation behavior as compared to the highly ductile deformation behavior of MCC. This brittle deformation causes smaller contact areas between primary particles for DCP than between MCC particles. Nonetheless, CS experiments applying the rotary press profiles display the same trend and tensile strengths are comparable to the rotary press results (Figure 2). Due to its abrasive nature, pure DCP was always studied admixed with 1 wt.-% of MgSt and is accordingly discussed in Section 3.3.

Pure tableting excipient without visco-plastic behavior and without the addition of other functional excipients (e.g., lubricants) can accordingly be very well transferred from CS to pilot and production scale rotary presses.

### 3.2. Effect of Compaction Profile

**Figure 3:**
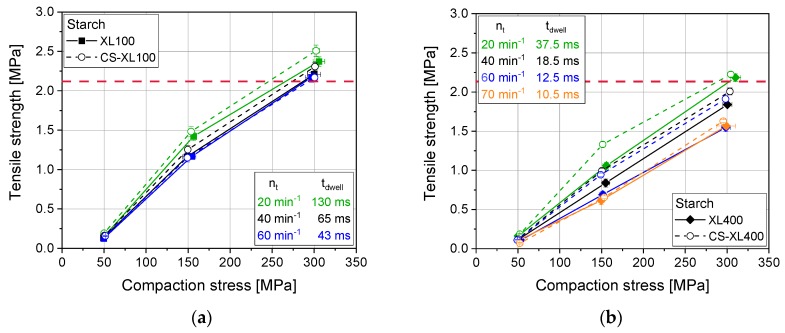
Tabletability profile for starch on XL100 (**a**) and XL400 (**b**) as well as for their simulation profiles applied on a CS. Red dashed line presents a guide to the eye for comparable results at comparable dwell time (approx. 40 ms) at approx. 300 MPa on both rotary press scales, applying 14 mm punches, respectively. *n* = 10.

Materials with visco-plastic deformation behavior pose a higher challenge towards the precision of the scaling of tableting processes in general and of compaction simulations in particular. Starch for instance displays a highly speed-dependent compaction behavior [5,32]. At high stress rates low deformation results while at low stress rates higher deformation and by that lower porosities and higher strengths result at the same compression stress. The transfer from lab to production scale for tablet formulations containing such materials bears risks as the compaction and the dwell times on production presses are significantly lower than those on lab rotary presses (Figure 3). Accordingly, the tensile strength of the resulting tablets is also speed-dependent and yields much lower values for quicker processes at production scale, as also described in literature [33]. For the simulation of these different scales via CS, the same trend towards higher tensile strengths at higher dwell times could be confirmed. This makes it possible to generally study the compaction speed-dependent effect of rotary presses on a CS. However, a slight, but consistent general trend to higher tensile strengths of CS-produced tablets as compared with tablets of the original rotary press, produced at the same (simulated) speed, was found (Figure 3).

**Figure 4:**
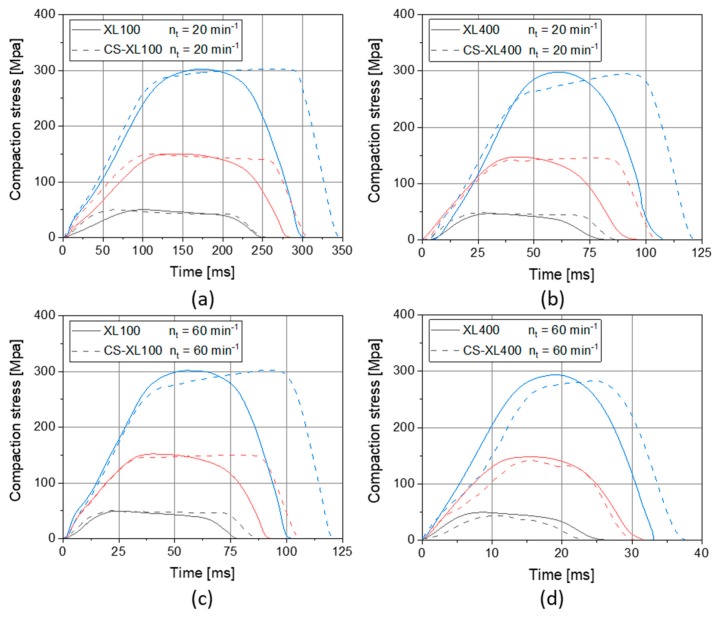
Comparison of compaction profiles of rotary presses and their respective simulation by CS for XL100 at 20 min^−1^ (**a**) and at 60 min^−1^ (**c**) as well as XL400 at 20 min^−1^ (**b**) and at 60 min^−1^ (**d**), at different target compaction stress of 50 (black lines), 150 (red lines), and 300 MPa (blue lines), applying 14 mm punches, respectively.

This phenomenon was traced back to the slight differences between the rotary press profile and simulated compaction profile on the CS (Figure 4). The stress over time profiles display that the compaction performed by the CS is slightly prolonged as compared with the original rotary press profile. The extent of prolongation was found to be dependent on the compression speed as well as on the compression stress. The difference is enhanced at elevated stresses and reduced at high speeds. This may be based on specific differences in punch movement. Such a prolongation of up to 20% while keeping the same maximum compaction stress explains the systematic deviation of tensile strength to higher values for starch in CS results. These differences in curve progression lead to structural differences in tablets of compression speed and dwell time-dependent materials such as starches. This additionally causes altered tablet properties such as tensile strength, as also described in literature [5,32].

**Figure 5:**
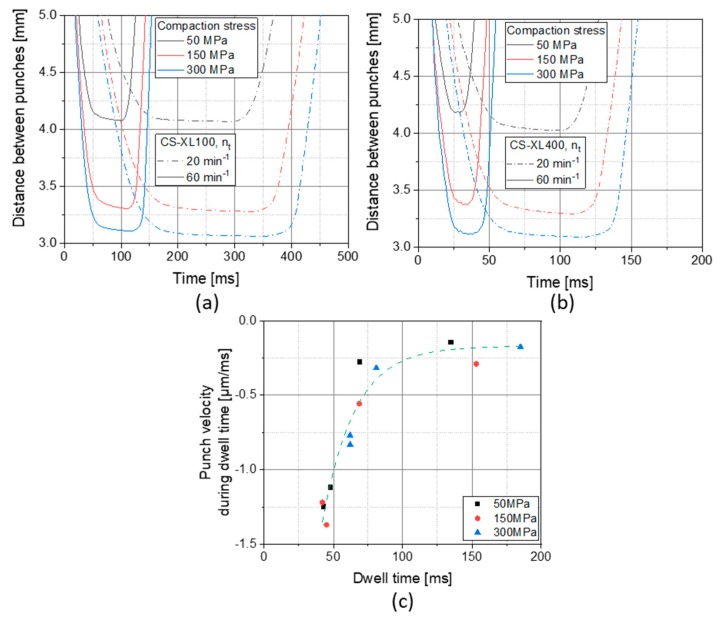
Effects of compaction stress, simulated turret speed and rotary press scale on the distance between punches over time profiles on the CS, simulating an XL100 at 20 and 60 min^−1^ (**a**) and an XL400 at 20 and 60 min^−1^ (**b**) as well as the correlation of the residual velocity of the punches during dwell time with the dwell time for the CS (**c**). All data refer to compression of starch. Negative punch velocity values indicate a further approximation of the 14 mm punches during dwell time.

An additional finding is that the compression stress is unexpectedly rising during the dwell time in CS experiments. This phenomenon is highly uncommon as punches reach a steady minimal distance over the period of time the plateau of the punch head is passing under the lowest point of the compression rollers in rotary presses. It is common that the stress is slightly falling during the dwell time which can be seen for rotary press results and CS results at low speed (Figure 4a) and at low pressure (Figure 4a–d). This can rationally be explained by the deformation of the material over time that reduces the stress in the material and by that, as the distance between the punches remains the same, reduces the measured compression stress. The phenomenon of rising stress during dwell time accordingly cannot originate from the material deformation behavior, but can be traced back to the distance between punches over dwell time (Figure 5).

It is visible that the distance between punches reduces over dwell time in CS experiments. This behavior is more pronounced with higher compression speeds. Only at the highest compression speed, simulating an XL400 at 60 min^−1^, this effect seems to level out as the dwell time becomes short (theoretically 12.5 ms) and noise originating from machine vibration disturbs proper evaluation (Figure 5b). Fitting the distance between punches linearly over dwell time results in the remaining punch velocity during dwell time that is plotted in the Figure 5c. It is obvious that the remaining punch velocity correlates with the dwell time and depends to a much lesser extent on the compaction stress. At lower dwell times, a higher residual punch speed results and accordingly explains the findings of rising stresses over dwell time (Figure 4).

These phenomena are based on the interplay between material properties and machine control. In general, bulk deformation is easily performed in not-highly-compressed systems. The more material systems become compressed, the higher stresses must be exerted to achieve a certain deformation. Accordingly, material deformation compensates the residual punch movement at low stresses and by that low confinement of the bulk particles. At higher stresses and by that higher compression and particle confinement levels, the bulk is less able to deform and reduce stresses and the same residual punch movement as at low stresses will result in compression stresses rising over dwell time. The residual punch movement is most likely due inertia in the fine control of the CS, which may be due to either electrical control, programming, or the inertia of the mass of the machinery. The latter may be the most likely explanation, as one major construction difference between CS and rotary presses is that on the CS much higher masses are moved vertically than on rotary presses. When on rotary presses only the punches are accelerated and hit the bulk material, a whole setup of electrical motor, toothed belt, screw gear, guides and punches must be accelerated in the CS and is decelerated by the bulk material. This difference might also aid the interpretation of noise level differences between rotary press and CS as seen at high tableting speeds (Figure 4d).

As a general outcome, it can be concluded that the kinetics or time-dependence of deformation behavior for the pure materials and the tableting mixture should be considered or directly analyzed in the course of process and formulation development. If a kinetic-dependent deformation behavior (i.e., strain rate or dwell time-sensitive) is found, special attention must be paid to the compression profile of the rotary press and the respective CS simulation. As the compression and dwell times were found to be influenced by the maximum compaction stress to a higher extend on the CS than on the rotary press, also these differences should carefully be checked to achieve a precise scaling approach between CS and rotary presses.

### 3.3. Effect on Lubricated Powders

**Figure 6:**
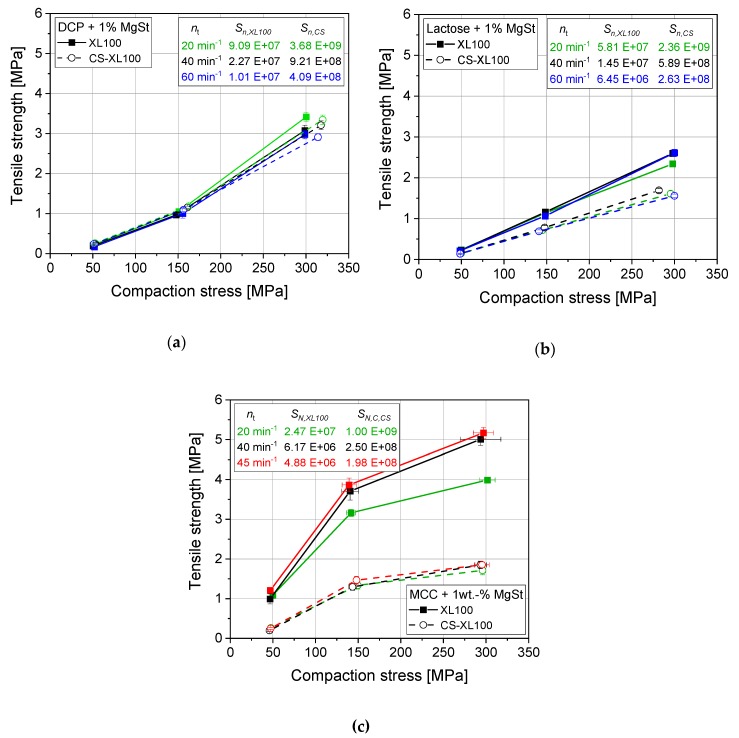
Comparison of tabletability profiles of DCP + 1 wt.-% MgSt (**a**), lactose + 1 wt.-% MgSt (**b**), and MCC + 1 wt.-% MgSt (**c**) on an XL100 as well as its simulation profiles applied on a CS with different (simulated) turret rotation frequencies, applying paddle rotation frequencies of n_p,XL100_ = 20 min^−1^ and n_p,CS_ = 272 min^−1^ to achieve convenient filling, applying 9 mm punches. *n* = 10.

**Figure 7:**
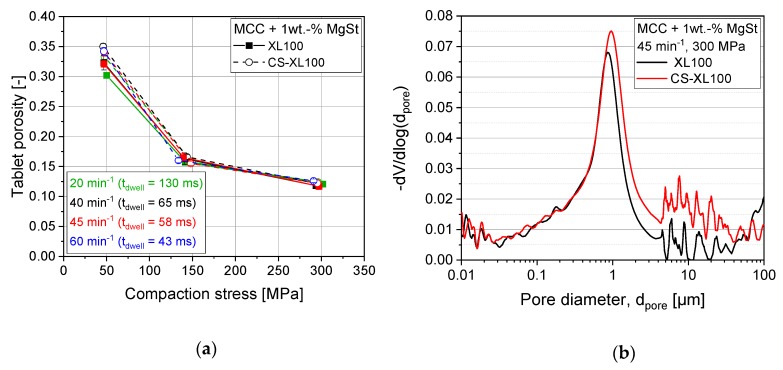
Effect of compaction simulation on porosity (**a**, *n* = 10) and pore size distribution (**b**, *n* = 1) of MCC + 1 wt.-% MgSt tablets.

Most pharmaceutically marketed tablet formulations contain lubricants to facilitate easy and robust production processes, avoiding unacceptable ejection forces, sticking to punches, and excessive wear of tooling [25,29,34]. Lubricants are most commonly admixed to the powder formulations as particulate additives with MgSt being the most frequently applied lubricant. Studies with binary mixtures of filler and lubricant were performed on the CS with feeder settings that showed a convenient filling behavior. They displayed good correlation of CS results to rotary press results for DCP, the most brittle excipient (Figure 6a). For intermediately deforming lactose, a discrepancy between rotary press results and its simulation was found (Figure 6b). This discrepancy was even more pronounced for MCC, showing mainly ductile deformation behavior. For lactose, the CS yielded approx. 65% while for MCC it only yielded approx. 35% of the tensile strength compared with those resulting from rotary press experiments (Figure 6b–c). Although the tensile strength dropped drastically in compaction simulation, the resulting tablet porosity and pore size distribution were found not significantly altered for MCC (Figure 7). These findings illustrate that the cause of lower tensile strength is not the resulting pore structure, but that the dispersion and distribution of lubricant particles with and onto excipient particles must be altered in the CS process as compared with the rotary press. A reduction in tensile strength was also found for results of the XL100 rotary press with decreasing turret speeds for MCC (Figure 6c). This cannot be related back to visco-plastic effects as these would not occur at such comparably long dwell times, would especially cause lower tensile strengths at higher compaction speed, and did not occur for pure MCC (Figure 1). Accordingly, this hints at an effect causing tensile strength reduction that correlates with the residence time within the feeder as it is also reduced at higher turret rotation frequencies.

**Figure 8:**
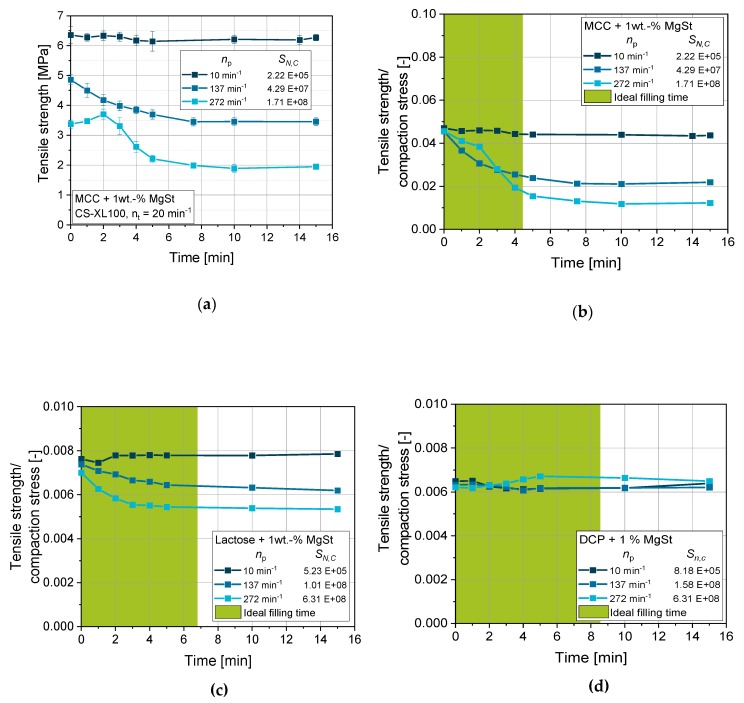
Effect of feed paddle speed and runtime of the CS (simulating an XL100 at 20 min^−1^ turret speed) on the tensile strength of lubricated (1 wt.-% MgSt) excipients: not normalized for MCC (**a**) and normalized to compaction stress for MCC (**b**), lactose (**c**), and DCP (**d**), applying 14 mm punches, respectively. *n* = 10.

The distribution of particles in powder blends is determined by the blending process [22] before feeding the mixture to the tableting process, but also by the feeding system of the tablet press itself [35]. The CS applies the powder (blend) by means of a paddle feeder, which is generally mimicking the powder feeding process on rotary presses. To differentiate the influence of the feeding process on the CS from effects of prior blending, time-dependent tensile strength studies were performed. To this end, pre-blended mixtures were applied to the paddle feeder and the tableting process was started, compressing the powder at approx. 150 MPa and taking samples over the runtime while filling depth and minimum distance between punches during compaction was kept constant. Tensile strength results plotted over runtime for different feeder speeds displayed that the tensile strength is dependent on paddle feeder speed for MCC (Figure 8a). The starting conditions seem impaired for different paddle speeds as the tensile strengths start from different values. This can be explained by the fact that the paddle speed not only determines the dispersion efficiency of lubricant particles but also the filling efficiency of the die. In this case, higher paddle speeds yielded a less efficient filling of the die as the filling depth was kept constant. A lower filling mass also causes lower compaction stresses when the tablet press is set to reach a fixed minimum distance between the punches in the compaction phase, being the common procedure (and mechanical setup) of most rotary presses. To exclude this effect from interpretation, the tensile strengths were normalized by the maximum compaction stress reached for the respective tablets. This assumes a good correlation of tensile strength and compaction stress in the questionable range. By applying this correction, a common starting point for all paddle feeder speeds is displayed, excluding the effect of filling efficiency and taking only the effect of lubricant dispersion and distribution on the mechanical weakening of the tablet structure into account (Figure 8b).

Higher paddle speeds cause a more drastic decrease in tensile strength of up to 70% between 10 and 272 min^−1^ paddle wheel rotation frequency for lubricated MCC (Figure 8b). This can be traced back to the higher specific energy input and by that more efficient dispersion of the MgSt particles, causing them to cover the surfaces of the excipient particles [22,36]. This lessens the bonding forces within the tablet structure as lubricants show lower bonding forces when present at the interface of particles than excipient particle contacts themselves [21]. The effect is less pronounced when the deformation behavior is altered from ductile for MCC to intermediate (ductile/brittle) for lubricated lactose (Figure 8c). In the case of lactose, a loss of approx. 30% between 10 and 272 min^−1^ paddle wheel rotation frequency is yielded. For both cases MCC and lactose, tensile strength reaches a plateau in the range of the calculated ideal filling time (calculated according to Section 2.2) in the feed frame, displaying that an equilibrium of influx, efflux, and dispersion is reached in this rough estimate for the mean residence time.

For the highly brittle and stiff material DCP, the influence of the paddle speed and by that the dispersion of the lubricant is not detectable (Figure 8d). In case of brittle materials, a high extent of new surfaces is generated due to particle fracture during the compaction. These newly provided surfaces are free of lubricant particles and the tablet structure accordingly develops a high number of contacts that are not weakened by the inclusion of lubricant particles [37,38].

To successfully scale tableting results from CS to rotary presses for the transfer to production or from rotary presses to CS for trouble shooting or re-formulation, the properties of the bulk material blend to be tableted and the effect of shear stresses during the tableting process on its tabletability and compactibility must be taken into account. Without an extensive characterization of the bulk material to be tableted, the shear stresses that are exerted to the bulk materials must be kept perfectly constant between all scales.

### 3.4. Consideration of Process Differences

To clarify the diverging results on rotary presses and CS, the stressing of the powder blend in the feeding system and by that the differences in layout and operational principle of either machine must be considered. On rotary presses, the feeder is stationary while the die table is moving, withdrawing powder from the feeder. The rotation mode of the feeder paddle wheel and the die table is continuous and by that the stressing of the powder blend within the tableting machine is only time-dependent and can be estimated by the shear number introduced by Narang et al. [26]:

(3) $$S_{N}=\left(\frac{\pi d_{p}n_{p}}{c}\right)\cdot \left(k_{s}n_{p}\right)\cdot {\left(\frac{m}{wn_{t}k_{d}}\right)}^{2}=\left(\frac{\pi d_{p}k_{s}}{c}\right)\cdot {\left(\frac{m}{wk_{d}}\right)}^{2}\cdot {\left(\frac{n_{p}}{n_{t}}\right)}^{2}$$

where $d_{p}$ is the diameter of paddle wheel, $k_{s}$ is the number of spokes, c is the clearance between spokes and base of the feeder, m is the powder mass in the feed frame, w is the weight of one tablet, $k_{d}$ is number of dies, $n_{p}$ is the rotation frequency of the feeder paddle, and $n_{t}$ is the rotation frequency of the die table. This approach combines the shear rate/intensity $\frac{\pi dn_{p}}{c}$ with the shear frequency $k_{s}n_{p}$ to yield an overall measure for shear stresses per time unit. This is further combined with the squared residence time estimated by ${\left(\frac{m}{wn_{t}k_{d}}\right)}^{2}$.

**Figure 9:**
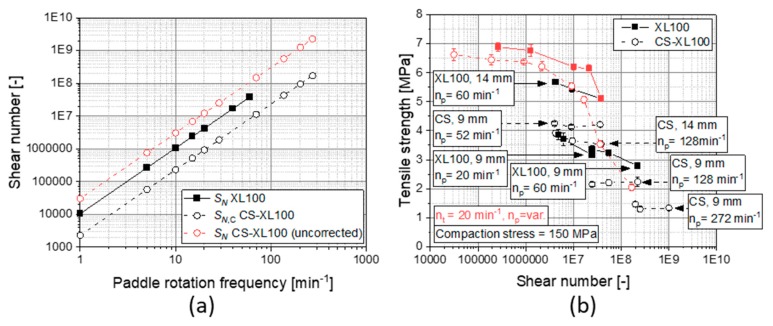
Comparison of shear numbers for an XL100 and uncorrected and corrected shear number for the CS simulating the XL100 at a turret rotation frequency of 20 min^−1^ and 9 mm punches (**a**) as well as the effect of shear number on the tensile strength, elucidating the influence of turret rotation frequency, paddle rotation frequency, and punch size on this correlation on an XL 100 and on a CS simulating the XL100 (**b**, *n* = 10). All data and experiments for MCC admixed with 1 wt.-% of MgSt.

In this approach for rotary presses, all processes that are taken into account are based on rotation events (feeder paddle and turret) and their timely proportions are defined by the angle which a specific component occupies on the die table. Accordingly, the time available for filling the die is determined by the filling angle $\varphi_{\mathrm{fill}}$ of the feeder divided by the rotation frequency of the turret. Additionally, the mass flow rate can also be directly determined by $wn_{t}k_{d}$, assuming constant tablet weight. When applying this approach straight forward parametrically to the setting of the CS, a much too high shear number would be calculated (Figure 9a). Hence, a thorough assessment of the influencing machine parameters and the differences in the mode of operation becomes important.

Besides layout differences (such as the diameter and clearance of the feeder paddle) that are directly integrated in Equation (3), the operation mode of the CS used in this study is different, providing a stationary press station and a moving paddle feed frame. The CS is especially mimicking the compaction profile, while also keeping the filling time and mode for the simulated rotary press as realistic as possible, e.g., also performing suction filling by moving down the lower punch underneath the feeder powder bed. Nonetheless, the stressing of the powder is generally different to a rotary press feeder due to two facts: (1) The paddle wheel of the CS is only actuated when the feeder is placed over the die; (2) there is only one punch underneath the feeder of the CS while there are several dies under the opening of the feed frame of common rotary presses, which influences the mean residence time and by that the stressing time in the system. Accordingly, these differences must be accounted for by including adaptions in the shear number approach. For the actuation pattern of the feeder paddle of the CS (1), the correction factor must take into account that the feeder paddle is only actuated during the respective filling time of the dies on the rotary press. As a consequence, the rotation frequency of the paddle $n_{p}$ must be corrected by the share of the fill angle in the full circle $\frac{\varphi_{\mathrm{fill}}}{360^{{}^{\circ}}}$. The fact that only one die is under the feed frame for the CS while usually multiple dies are under the powder bed in the feed frame of rotary presses (2) must be taken into account too. This setting enhances the shearing of the powder compared with the rotary press setup because the powder available to fill the (only one) die on the CS is sheared multiple times before it flows into the die. To correct this additional shearing, the rotation frequency of the paddle $n_{p}$ is corrected by the relation of the fill angle to the angle between the dies $\frac{\varphi_{\mathrm{fill}}}{\varphi_{\mathrm{dies}}}$ of the original rotary press to be simulated, respectively. Including these correction terms into the shear number equation of Narang et al. ([26], Equation (3)) yields Equation (4) to be applied for the CS used in this study:

(4) $$S_{N,\;C}=\left(\frac{\pi d_{p}k_{s}}{c}\right)\cdot {\left(\frac{m}{wk_{d}}\right)}^{2}{\left(\frac{n_{p}}{n_{t}}\cdot \frac{{\varphi_{\mathrm{fill}}}^{2}}{360^{{}^{\circ}}\cdot \varphi_{\mathrm{dies}}}\right)}^{2}$$

By calculating the shear numbers for experiments presented in Figure 6 that were performed by intuitively choosing process parameters for convenient filling, it becomes obvious that the shear number is approx. two magnitudes higher on the CS as compared to the rotary press. This directly explains the drastic loss in tensile strength seen on the CS as a function of the ductility of the materials. Accordingly, it is highly necessary to account for the shear intensity in the scaling of tableting processes between CSs and rotary presses.

General studies taking the effect of turret rotation frequency, paddle rotation frequency, and punch size on the tensile strength of tablets of MCC + 1 wt.-% MgSt into account are displayed in Figure 9b as a function of shear number. The general trend of a drastic loss of tensile strength between shear numbers of 1E6 and 1E9 is met on both systems CS and rotary press. Specifically the paddle rotation frequency appears to be crucially determining the tensile strength, while the effect of the alteration of the turret speed has a lesser extent of influence on the tensile strength when compared by the respectively calculated shear number. This means that the shear intensity/shear rate ,dependent on $n_{p}$, is more determining than the cumulative shear energy input to the powder which is also determined by $n_{t}$. The size of the punches used also plays a role. Although reaching nominally comparable shear numbers, lower tensile strengths result with a smaller punch size. This might display that the shear number equations, in turn, underestimate the influence of the residence time. Other possible explanation are the special shearing of powder during dosing-out of surplus powder after filling, which occurs much more often with smaller punches as well as the wrong estimation of the residence time by $\frac{m}{wn_{t}k_{d}}$ because the fill level in either feed frame may vary also dependent on the particle and flow properties of the powder.

**Figure 10:**
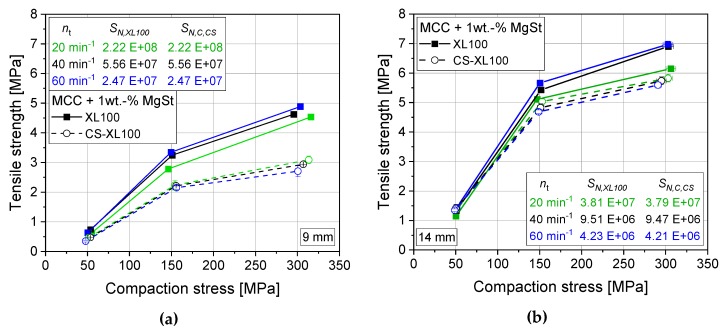

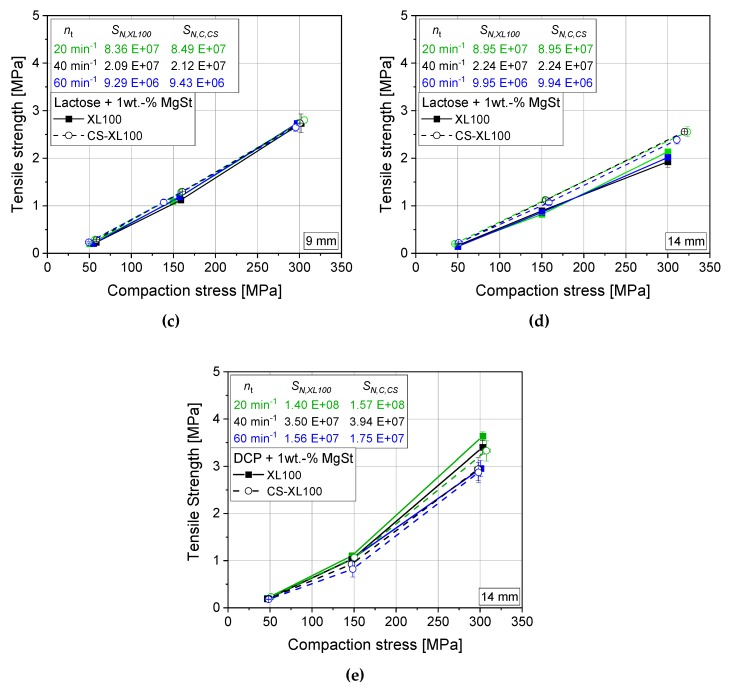
Tabletability profile of lubricated powders (1 wt.-% MgSt) for an XL100 and its simulated profile (CS) by applying optimized feeder rotation frequency using the CS-adapted shear number $S_{N,\;CS}$ for MCC applying 9 mm punches (**a**, n_p,XL100_ = 60 min^−1^, n_p,CS_ = 128 min^−1^) and 14 mm punches (**b**, n_p,XL100_ = 60 min^−1^, n_p,CS_ = 128 min^−1^), lactose applying 9 mm punches (**c**, n_p,XL100_ = 24 min^−1^, n_p,CS_ = 52 min^−1^) and 14 mm punches (**d**, n_p,XL100_ =60 min^−1^, n_p,CS_ = 128 min^−1^) as well as DPC applying 14 mm punches (**e**, n_p,XL100_ = 60 min^−1^, n_p,CS_ = 128 min^−1^). *n* = 10.

For testing its practical application, the $S_{N,\;C}$ can be used as an estimator to predefine the correct rotation frequency of the CS feeder, based on the $S_{N}$ for the corresponding rotary press. By applying this approach and using approx. equal shear numbers, tensile strengths of the highly sensitive mixture of MCC and 1 wt.-% MgSt are brought closer to the results achieved on the rotary press (Figure 10a–b). However, differences are still obvious. Especially for small punches of 9 mm, lower tensile strengths result for the CS (approx. 35% lower) compared with larger punches of 14 mm that only yield approx. 15% deviation always to values lower than those of the rotary press. For lactose, a clear influence of the punch size can be seen as well with larger punches again yielding higher tensile strengths (Figure 10c–d). Nevertheless, the tensile strength values for lactose are perfectly matched for 9 mm punches while the CS yields higher tensile strengths as compared with the rotary press for 14 mm punches. DCP on the contrary, remains unsusceptible to process parameter changes in general and the shear number specifically, as it shows comparability of tensile strength over a wide range of turret speeds, paddle rotation frequencies, and punch size (Figure 6c; Figure 10e).

This drastic effect of material properties and punch size makes a further improvement of the shear number approach necessary to be holistically applicable for scaling tableting processes. Probably, material properties such as flow and densification properties under low stresses are of significant importance, also for realizing a precise scaling from CS to rotary press. It can further be concluded that process parameters that appear to be of secondary importance at the first glance must also have a pronounced influence on tablet tensile strength of lubricated powders that are susceptible to overlubrication. Such process parameters may be the overfilling of dies and the dosing-out of excessively filled powder that is pushed back into the feeder, but might experience a different shearing history on the different types of machines. This hypothesis is further fostered as the overview of the variation of different process parameters (Figure 9b) shows that the shearing intensity (directly correlated with the paddle rotation frequency and the clearance between spokes and bottom of the feed frame) has a more pronounced influence on tensile strength than the residence time (mainly defined by punch size/tablet mass and turret speed). That only the size of the punches has this effect is by trend disproved by the fact that DCP displays the same tensile strength for both punch and by that tablet sizes (Figure 6c; Figure 10e). Additionally, the interplay of material flow properties and process parameters can also vary the filling level in the feed frame and this would in turn alter the residence time as well as the density of the bulk powder to be transported in the feed frame and filled into the dies. Accordingly, these phenomena need to be investigated in depth and recorded in figures in future research.

## 4. Conclusions

The simulation of rotary presses on a compaction simulator straight forwardly provides good agreement with original rotary press results if no challenging materials or blends are processed. In case of visco-plastic deformation behavior, the accordance of the simulated compaction stress over time profiles must carefully be checked to avoid systematic deviations in tensile strength and by that in scaling precision. For lubricated blends, the specific differences in layout and operation mode between rotary press and compaction simulator must be considered. By thorough process investigation, the shear number equation was extended and adapted to be applied on the CS used in this study. With the presented approach, challenging formulations, that also display trends, e.g., overlubrication in production scale, can better be assessed on compaction simulators and transferred to production rotary presses or, vice versa, production challenges can better be transferred back to lab scale on a CS for root cause investigation and reformulation.

Regarding the simulation of rotary press processes on compaction simulators, the feeding remains a key challenge to be further investigated as it crucially determines production capabilities (i.e., capacity limits, secure parameter space for tablet production) and final product properties. The modified shear number approach must further be intensified to more precisely account for material properties and specific process parameters such as overfilling. Furthermore, it should be adapted to other compaction simulators as well. A future challenge is the prediction of the filling success by means of compaction simulators or other methods and mathematical models to enable direct prediction of production capacity using a compaction simulator. This could also support general production process chain decisions by elucidating the effect of interposed granulation steps.
